# Nondrainage after Laparoscopic Cholecystectomy for Acute Calculous Cholecystitis Does Not Increase the Postoperative Morbidity

**DOI:** 10.1155/2018/8436749

**Published:** 2018-07-02

**Authors:** Jianguo Qiu, Ming Li

**Affiliations:** Department of Hepatobiliary Surgery, The First Affiliated Hospital of Chongqing Medical University, Chongqing 400016, China

## Abstract

**Background:**

It is now established that prophylactic drainage is not needed after laparoscopic cholecystectomy (LC) for chronic calculous cholecystitis. However, the benefit of drains versus their potential harm for acute calculous cholecystitis (ACC) following laparoscopic LC has been questioned. Therefore, we conducted a comparative study to assess the need for drainage.

**Methods:**

Between January 2014 and October 2016, 212 patients with ACC undergoing LC undergo either drainage (n= 106) or no drainage (n= 106). The primary end points were the number of patients with postoperative drain-related complications, early and late Visual Analogue Scale (VAS) score, and hospital stay. Secondary end points included estimated blood loss, postoperative recovery, analgesia requirement, and cosmetic satisfaction result.

**Results:**

There was no bile duct injury and mortality in both groups. The overall complication rate was 12.5% with no significant difference between those with or without drainage (*P*=0.16). Normal activity resumption was significantly faster and the postoperative hospital stay was slightly shorter in the nondrainage group (*P *=0.03 and* P*= 0.04, respectively). The early VAS score in the drainage group was significantly higher (*p< *0.05). There were no significant differences between the two groups in postoperative hematology test, late VAS score, and patient satisfaction of cosmetic outcome.

**Conclusion:**

Routine drainage for patients with ACC after LC may not be justified with similar drain-related complications compared with nondrainage group.

## 1. Introduction

The evolution of laparoscopic techniques has transformed traditional surgery to a considerable extent. Compared with an open approach, laparoscopic cholecystectomy (LC) has become the gold standard technique for uncomplicated and complicated cholecystitis [1–3]. Prophylactic drainage of the peritoneal cavity after gastrointestinal surgery has been widely practiced to prevent intraperitoneal fluid collections and to detect early complications, such as postoperative hemorrhage and leakage of bile [4, 5]. However, with the development of laparoscopic surgery and advancement of surgical techniques, prophylactic drainage of the abdomen after surgery has since been questioned.

Several trials have shown that drains were of no benefit after gastrectomy [4], hepatic resection [6], splenectomy [7], pancreatic resection [8], and colonic resection [9] as well as elective LC for uncomplicated cholecystitis [1, 2]. It seems that drainage does not prevent postoperative complication. Instead, drainage-related complications such as fever, wound infection, wound hernia, or hemorrhage may cause unnecessary discomfort to patients [4, 5, 10].

There is census that drainage should not be considered mandatory or standard after elective LC. To our knowledge, however, limited information is available on routine prophylactic drainage after LC for patients with acute calculous cholecystitis (ACC). Therefore, we hypothesized that the use of drain during LC for ACC patients is not beneficial and that the routine drainage of gallbladder bed after LC may not be justified. To test this hypothesis, we conducted a cohort study in a population of ACC patients undergoing LC comparing the outcomes, between those received drainage and those without drainage.

## 2. Patients and Methods

### 2.1. Study Period and Patient's Population

**Figure 1:**
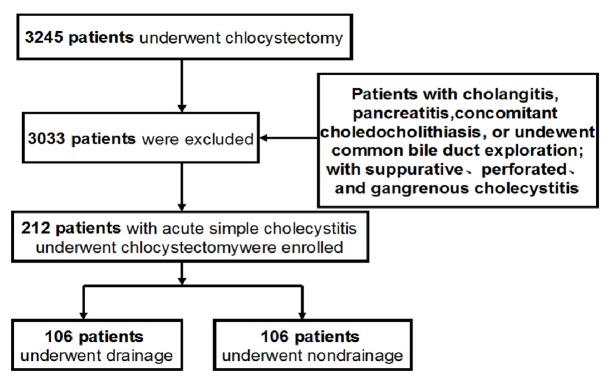
Flowchart of patient recruitment.

This study was performed under a human investigational protocol that was approved and monitored by the Institutional Review Board of our hospital. A prospectively maintained database consisting of a consecutive series of 212 patients with ACC aged 20 years and older was submitted to undergo LC from January 1, 2014, and October 31, 2016, and was used in current study (Figure 1). Patients with cholangitis or pancreatitis were not included. Patients with evidence of concomitant choledocholithiasis were treated with preoperative endoscopic retrograde cholangiopancreatography (ERCP) and/or common bile duct exploration; patients with suppurative, perforated, and gangrenous cholecystitis; and patient who is unwilling to participate also were excluded.

### 2.2. Study Design

According to whether a prophylactic drain was inserted or not during the operation, patients were allocated into two groups: group A for patients with drain implantation during operation and group B for patients without drainage. We set the number of patients with postoperative drain-related complications, early and late VAS score, and hospital stay as the primary end points; estimated blood loss, postoperative recovery, and cosmetic satisfaction result as the secondary end points.

### 2.3. Surgical Procedure

LC was performed by three experienced surgeons and all patients were performed by total cholecystectomy. The standard surgical procedure was the same as previously described [12, 13]. If necessary, a modification of the operative technique was used to facilitate the surgical procedure, including gallbladder decompression, use of sutures to control cystic duct, use of endoscopic pouches to retrieve specimen, and enlargement of subumbilical incision as well as use of a fifth port. If drainage was performed, a drain tube made of polyethylene was placed at the end of the LC (in patients selected by draw) through the trocar (5 mm) at the anterior axillary line. The drain tube was in place for at least 24 hours, and it was removed the first postoperative day usually except abdominal distension, drainage more than 20 mL/d, and residual peritoneal fluid detected in postoperative ultrasonographic findings. The protocol of the postoperative analgesia was the same for all the patients. Two doses of Tramadol Hydrochloride (50 mg) every 12 hours were prescribed to all patients postoperatively. All patients were given fluids 8 hours postoperatively unless there was nausea or vomiting. The second day, a fat-free diet was given. After discharge, all patients returned for examination on the 7 th and 60 th postoperatively.

### 2.4. Definition

ACC was defined as gallstone according to ultrasound examination, abdominal pain, tenderness in the right upper quadrant, and a temperature of more than 38°C. Postoperative pain was evaluated on postoperative 6, 12, 24, 48, and 72 hours and 1 week. A standard 10-cm VAS with options ranging from 0 (no pain) to 10 (worst pain) was used to assess postoperative pain scores. Early postoperative pain was assessed 6 to 48 hours after surgery, whereas late postoperative pain was assessed from 72 hours to 1 week after surgery. An index scored from 0 (no satisfaction) to 10 (complete satisfaction) was used to assess the cosmetic satisfaction result. Complications or death occurring during the same hospitalization or within 30 days after operation were defined as postoperative complications and postoperative mortality, respectively. Operating time was the number of minutes for which the operation continues. Hospital stay was defined as the number of days from the date of operation to discharge. All reported complications were reclassified as suggested by Dindo et al. [11].

### 2.5. Data Collection and Statistical Analysis

Preoperative, perioperative, and postoperative data for all patients were recorded continuously according to protocol prospectively. Differences between the 2 groups were analyzed by the Mann–Whitney U for continuous variables, and the categorical variables were analyzed by the *χ*2 test or continuity correction method. All statistical tests were 2-sided, and a significant difference was considered when* P *< 0.05. The statistical analyses of the data were performed using the SPSS 16.0 statistical software.

## 3. Results

### 3.1. Study Characteristics

**Figure 2:**
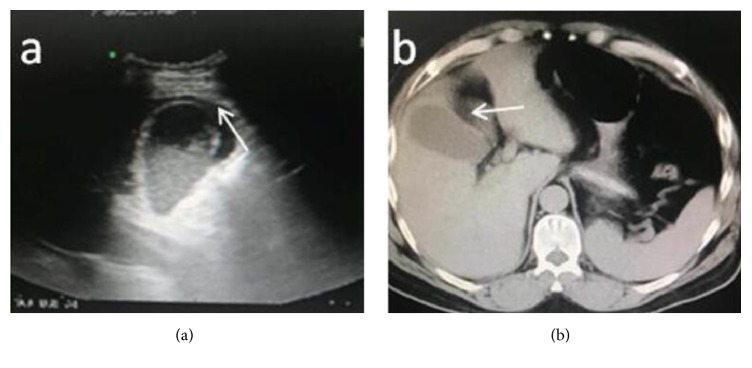
Preoperative abdominal ultrasonography and CT scan indicate pericholecystic collection (white arrow) in a patient on admission.

In total, 212 patients with ACC underwent LC were included in this study: 106 in the nondrainage group (group A) and 106 in the conventional drainage group (group B). ***Significantly, more patients with pericholecystic collection (Figure 2) were found to be in the drained group [22 (20.7%) versus 14 (13.2%); P<0.01]***. The mean age of the drainage group was 45 years (range, 19-75 years), which did not significantly differ from the 43 years (range, 20 -74 years) for the nondrainage group (P = 0.44). There were no differences between the 2 groups in the distribution of sex, body mass index, American Society Of Anesthesiologists (ASA) score, and cyst size, respectively (Table 1). The ultrasonographic findings of the patients in the two groups were comparable, as shown in the Table 2.

### 3.2. Primary Endpoints

Neither group had intraoperative complications or required transfusion during or after surgery. There was no statistically significant difference between the drainage and nondrainage groups with regard to either postoperative complications or their severity (Table 3). The overall rate of patients having 1 or more complications eventually related to drainage was 12.5% (12/106 [11.3%] in the drainage group versus 14/106 [13.2%] in the conventional nondrainage group;* P *=0.16) respectively. There was no bile duct injury in both groups. Abdominal ultrasonography show subhepatic fluid collection in 7 patients (6.6%) in drainage group and in 9 patients (8.5%) in nondrainage group (*P *= 0.24) with median subhepatic collection was 25 mL (10–40 mL) in drainage group and 30 mL (15–50 mL) in nondrainage group (*P *= 0. 32). All subhepatic collections disappeared at 1-2 weeks after surgery examined by abdomen ultrasonographic examination. Several other postoperative complications including upper respiratory infection, urinary tract infection, wound infection, bowel ileus were also similar in both groups.

Pain score measured immediately after surgery in the recovery unit was lower in the nondrainage group than in the conventional drainage group (median, 8.8 versus 5.5;* P *<0.01). The postoperative VAS scores after 6, 12, 24, and 48 hours were lower in the nondrainage group with significance difference (Table 4). The postoperative late VAS scores after 72 hours and 1 week were similar in the two groups (*P *= 0.32 and* P *= 0.44, respectively). There were no deaths and no reoperations were needed. The average time to resume normal activity after surgery and the postoperative hospital stay of the nondrainage group were slightly shorter than those of the drainage group (Table 5; 2.0±0.8 versus 2.4±1.0 days and 3.0±1.4 versus 3.5±1.5 days, respectively).

### 3.3. Secondary End Points

There was no significant difference in the median estimated amount of operative blood loss between the conventional drainage group and the no drainage group (median, 75 mL; range, 30–250 mL) than in no drainage group (median, 70 mL; range, 20–300 mL;* P *=0.32). 12 patients suffered modified operative techniques without significant difference between the two groups (*P *=0.45). The mean operative time was slightly longer in drainage patients than in no drainage group (110 minutes versus 99 minutes, respectively) with statistics significantly (*P *=0.04). A marginally higher proportion of patients in the conventional drainage group received intravenous analgesia, and the median dosage of painkillers given was also higher in the drainage group compared with the no drainage group (Table 5). After discharge, all patients returned for reexamination on the 7th postoperative day and on the 90th day. There was no significant difference in the cosmetic result and overall patient satisfaction between drainage and nondrainage patients after 3 months follow up (*P *= 0.37).

## 4. Discussion

LC is one of the most common operations in gastrointestinal surgery [14]. Conventionally, a drain is routinely placed in the subhepatic region to monitor postoperative subhepatic fluid collection, bile leaks and bleeding [4, 5]. With the development of laparoscopic surgery and advancement of surgical techniques, the above complications have become less prevalent.

Numerous studies have shown that several abdominal surgical procedures can be safely performed without drainage [4, 6–9]. Drainage does not prevent complications; otherwise, increase the tube-related complications such as fever, wound infection, wound hernia, or discomfort to patients. Furthermore, the recent published randomized controlled trails and and meta-analysis performed by Picchio [15] and Bugiantella [16] were both mainly focused on the issue of the role of the drainage in elective or uncomplicated LC and concluded that there was no evidence to support the use of drain after this surgical procedure [4, 5, 10]. However, there are still limited data on the value of prophylactic drains following LC for patients with ACC. It is therefore we hypothesized that the use of drain during LC for ACC patients is not beneficial and that the routine drainage of gallbladder bed after LC may not be justified. For assessing the efficacy of nondrainage we conducted a prospective study to assess whether not using a drain will lead to increased mortality and morbidity. Our outcomes indicated that the overall morbidity of the nondrainage group was not significantly higher than that of the drainage group. Furthermore, drainage causes more postoperative pain and slightly longer hospital stay. These results were consistent with previous study after elective LC [17–19].

In the early years of laparoscopic surgery, ACC was considered a relative contraindication to LC due to a significant risk of complications [20, 21]. The rate of complications is mainly related to the severity of the gallbladder disease, such as the presence of suppurative, perforated, and gangrenous gallbladder inflammation [22]. Furthermore, conversion rate is relevant with a threefold increase when severe cholecystitis is present [22]; therefore, our study group only includes selected patients without severe cholecystitis. Compared with elective LC, the major challenge of LC for ACC patients is fluid collection. General morbidity and mortality are usually reported as worse when nondrainage instead of drainage is utilized after LC for patients with cholecystitis, this phenomenon was further confirmed by Gurusamy and his colleagues [17], which found that the total number of abdominal fluid collections is higher in the drainage group than in the nondrainage group after LC. However, statistical confirmation is not always achieved. In the current series, no statistical difference could be demonstrated when these variables were examined. LC for ACC has been associated with a higher conversion rate and a higher incidence of serious bile duct injuries compared with elective operations [23, 24]. It is therefore not surprising to find that critics have argued that these studies cannot be applied to ACC patients because the potential ramification of an undrained collection could be devastating to the patient [9]. Although the overall complication rates such us bile leakage, wound infection has diminished in recent years. However, before the current study, there were no randomized and comparative studies examining the role of intraperitoneal drainage after LC for ACC patients. In current series, there were no significant differences between the two groups with regard to the postoperative mortality and morbidity. Although the study was too small to determine whether nondrainage was truly responsible for ACC patients undergoing LC.

As shown in the literature, the clinical significant bile leak is a result of inappropriate surgical procedure or lack of drainage [25, 26]. Based on our experience, however, the drain does not ensure that postoperatively there will be no complication, unless the surgeons examine the patient frequently and thoroughly, as well as to anticipate omissions and mistakes during operation. Furthermore, the clinically significant bile leakage is very rare and it cannot be prevented by the use of a drain. The main objective of this study was to verify the hypothesis that routine intraperitoneal drainage is not required after LC for patients with ACC and demonstrated that LC without drainage had satisfactory results. However, there were several limitations that must be taken into account when considering these results in clinical application. Firstly, the overall methodological quality of this cohort study would to peer-heard. Secondary, there was no specific algorithm for applying drainage or nondrainage during LC in our center, thus whether drainage was selected to perform was left to the individual surgeon. This may introduce some selection biases. Thirdly, the sample size is too small to give confirm conclusions; Further prospective controlled studies are needed for a more comprehensive research on the efficacy of nondrainage after LC for AC patients.

In conclusion, LC without routine drainage is feasible and safe for ACC. Although the postoperative complications of LC without abdominal drainage were comparable with those patients with drainage, individuals undergoing nondrainage might benefit from a shorter hospital stay and a faster resumption without an increase in postoperative morbidity and mortality. However, due to the methodological deficiencies, further randomized controlled trials will be required in future study.

## Figures and Tables

**Table 1:** Clinical data and preoperative laboratory examination.

**Parameters**	**Drainage group** ***(n* = 106)**	**Nondrainage group** ***(n* = 106)**	**P ** **v** **a** **l** **u** **e** ^**∗**^
Age ( years)	45±12	43±14	0.44
Sex (M:F)	45/51	50/56	0.23
Body wight (kg/m2)	23.2±2.4	24.5±2.2	0.17
ASA status (I/II/III)	55/40/11	50/42/14	0.26
Previous biliary symptoms (n, %)	78 (73.6%)	80 (75.5%)	0.32
Duration of acute symptoms (h)	24.5±19.5	25.2±20.4	0.29
Duration of symptoms >3 days (n, %)	67 (63.2%)	70 (66.1%)	0.34
Previous abdominal surgery (n, %)	18 (16.9%)	15 (14.1%)	0.22
Fever > 37.5C (n, %)	75(70.7%)	77(73.3%)	0.19
Leukocyte count > 10X 109/L (n, %)	76(71.7%)	72(67.9%)	0.24
Laboratory examination			
Total bilirubin (umol/L)	21.7±3.5	22.5±4.8	0.15
Aspartate transaminase (U/l)	38.2±11.5	35.7±10.4	0.22
Alanine transaminase (U/l)	33.4±9.3	31.8±10.6	0.18
Alkaline phosphatase (IU/l)	110±23.7	100.5±25.1	0.14

^*∗*^Mann–Whitney U for continuous variables, and the categorical variables were analyzed by the *χ*2 test or continuity correction method.

**Table 2:** Ultrasound findings for the patients on admission.

**USG findings**	**Drainage** ***(n*=106)**	**Nondrainage** ***(n*=106)**	**P ** **v** **a** **l** **u** **e** ^**∗**^
Thickened gallbladder (n, %)	63 (60.3%)	59 (55.7%)	0.11
Edematous gallbladder (n, %)	90 (84.9%)	85 (80.2%)	0.24
Distended gallbladder (n, %)	88 (83.1%)	86 (81.1%)	0.28
Presence of gallstones (n, %)	96 (90.6%)	97 (91.5%)	0.32
USG Murphy^,^s sign positive(n, %)	64 (60.4%)	58 (54.7%)	0.27
Pericholecystic fluid (n, %)	22 (20.7%)	14 (13.2%)	0.01
Cyst size (cm)	10.5±3.3	9.7±3.1	0.21

USG: Ultrasonography; ^*∗*^Mann-Whitney U for continuous variables, and the categorical variables were analyzed by the *χ*2 test or continuity correction method.

**Table 3:** Postoperative complication according to Clavien-Dindo classification [11].

**Parameters**	**Drainage group** ***(n* = 106)**	**Nondrainage group** ***(n* = 106)**	**P value**
Total complications	16	15	0.15
No. patients with complications (n, %)	12 (11.3%)	14 (13.2%)	0.16
Bile leak (grade IIIa)	2	1	0.22
Fluid collection (grade I)	7	9	0.24
Bile duct injury (grade IIIa)	0	0	0.23
Pulmonary inflammation (grade II)	2	3	0.16
Incision hernia(grade II)	2	1	0.21
Bleeding (grade IIIa)	1	0	0.35
Wound infection(grade II)	2	1	0.21

According to Clavien-Dindo classification of surgical complications. Grade 1 complications required no surgical or medicinal treatment; Grade 2 complications required medicinal therapy; Grade 3 complications required surgical, endoscopic, or radiologic treatment. Grades 1 and 2 were considered as minor complications, whereas Grades 3-5 were considered as major complications.

**Table 4:** Postoperative pain score and cosmetic satisfaction results.

**Parameters**	**Drainage group** ***(n* = 106)**	**Nondrainage group** ***(n* = 106)**	**P ** **v** **a** **l** **u** **e** ^**∗**^
Early VAS pain score			
Immediate	8.8±0.6	5.5±04	< 0.001
6 hours	7.5±0.8	4.5±0.3	< 0.001
12 hours	6.8±0.5	4.0±0.5	< 0.001
24 hours	4.7±0.6	3.1±0.4	0.02
48 hours	4.5±0.4	2.5±0.2	0.03
Late VAS pain score			
72 hours	2.5±0.3	1.9±0.3	0.32
1 week	2.0±0.2	1.5±0.2	0.44
Cosmetic score	7.4±2.4	7.9±2.1	0.37

VAS: Visual Analogue Scale; ^*∗*^Mann–Whitney U test.

**Table 5:** Perioperative outcomes.

**Parameters**	**Drainage group** ***(n* = 106)**	**Nondrainage group** ***(n* = 106)**	**P ** **v** **a** **l** **u** **e** ^**∗**^
Modification of the operative technique (n, %)	24(22.6%)	26 (24.5%)	0.45
Operative time(minutes)	110±15.5	99±10.4	0.04
Blood loss(ml)	75±23.5	70±25.8	0.32
***Transfusion (u)***	***0***	***0***	
Dose of analgesic (mg)	105±50	75±25	0.02
Maximum gallstone size (cm)	2.0±1.1	1.9±1.3	0.55
Time to resume normal activity (days)	2.4±1.0	2.0±0.8	0.03
Hospital stay (days)	3.5±1.5	3.0±1.4	0.04

^*∗*^Mann–Whitney U for continuous variables, and the categorical variables were analyzed by the *χ*2 test or continuity correction method.
